# HIV-associated preeclampsia: evaluation of lymphangiogenesis in placental bed samples

**DOI:** 10.1007/s00418-025-02359-4

**Published:** 2025-05-22

**Authors:** O. A. Onyangunga, P. Naidoo, J. Moodley, T. Naicker

**Affiliations:** 1 Optics and Imaging Centre, Doris Duke Medical Research Institute, College of Health Sciences, University of KwaZulu-Natal, Durban, South Africa; 2 Women’s Health and HIV Research Group, Department of Obstetrics and Gynecology, School of Clinical Medicine, College of Health Sciences, University of KwaZulu-Natal, Durban, South Africa

**Keywords:** Early- and late-onset preeclampsia, Normotensive, Human immunodeficiency virus, Lymph angiogenesis, Lymphatic microvessel density

## Abstract

The role of angiogenesis in preeclampsia pathogenesis is widely studied; however, despite the lymphatic vessels’ complementary role to the blood vascular system, studies on their morphology in the placenta and placental bed are lacking. In total, 87 placental bed specimens were utilized, which were grouped into normotensive pregnant (*n* = 28), early-onset preeclampsia (*n* = 31), and late-onset preeclampsia (*n* = 28), and further stratified by human immunodeficiency virus (HIV) status. Tissue was immunostained with podoplanin antibody to investigate whether HIV infection affects lymphangiogenesis. The lymphatic capillary density and luminal areas within the placental bed were morphometrically assessed. Lymphatic microvessel density and mean area/lumen in the preeclampsia group were higher and larger than in the normotensive group, respectively (*p* = 0.01 and *p* = 0.001). A correlation between blood pressure levels and lymphatic microvessel density was observed (*r* ≥ 0.272; *p* ≤ 0.032). Significant differences were observed between the mean microvessel density of normotensive HIV-uninfected and HIV-infected groups (5.9 ± 2.3 versus 7.5 ± 2.8, *p* = 0.01) and late-onset preeclampsia HIV-uninfected and HIV-infected groups (7.1 ± 3.9 versus 7.8 ± 2.7, *p* = 0.01). The mean area/lumen between normotensive HIV-uninfected and HIV-infected, and late-onset preeclampsia HIV-uninfected and HIV-infected groups were significantly different (*p* = 0.03 and *p* = 0.001). Small lymphatic capillaries were significantly abundant in late-onset preeclampsia HIV-infected (*p* = 0.03) and normotensive HIV-infected (*p* = 0.0001) groups compared with uninfected groups. Lymphatic capillary density and area/lumen upregulation was observed in the placental bed of HIV-infected women, with a positive correlation between maternal blood pressure and lymphatic microvessel density, potentially affecting birth weight in the preeclampsia group.

## Introduction

A successful pregnancy depends on decidualization, implantation of the blastocyst, and the development of the placenta. Under steroid hormone regulation, decidualization is accompanied by significant remodeling of both blood and lymphatic vessels, ensuring the placenta receives an adequate blood supply (Yu et al. 2022). Preeclampsia (PE) is a pregnancy-specific disorder that accounts for substantial maternal and perinatal morbidity and mortality worldwide, being especially high in low- and middle- income countries (LMICs) (Ghulmiyyah and Sibai 2012; Moodley et al. 2016). This is a heterogeneous disorder that may be classified by gestational age into early-onset preeclampsia (EOPE), in which clinical signs and symptoms occur before 34 weeks of gestation, and late-onset preeclampsia (LOPE), where clinical signs and symptoms occurs after 34 weeks of gestation (Gathiram and Moodley 2020). They are considered two distinct disease entities, with severe complications of both the mother and baby occurring more often in EOPE compared with LOPE (Guo et al. 2021; Otalike et al. 2022). The placenta plays a significant role in the pathophysiology of PE (Chau et al. 2017; Jim and Karumanchi 2017).

Research on the vascular pathology of PE has focused on the role of uterine spiral arteries in the placenta and placental bed (Pijnenborg et al. 2010; Ergen et al. 2018). Coelho et al. (2006) reported decreased microvessel density in the decidua and myometrium in PE, with severe features associated with low birth weight babies (Coelho et al. 2006). In contrast to the role of angiogenesis in the pathogenesis of PE, studies on lymphatic vessel biology are sparse, despite their complementary role to the blood vascular system.

The lymphatic system is an essential vascular network for lipid absorption, fluid homeostasis, and immune surveillance (Cifarelli and Eichmann 2019). The lymphatic microvessels (LMVs) have a homeostatic role in the collection of arteriolar fluid leakage and drainage to collecting lymphatic vessels and nodes (Zhou et al. 2024). To accomplish this function, the LMVs undergo dilation, similar to spiral arteries, during pregnancy (Wang et al. 2011). Recently, He et al. (2017) and Windsperger et al. (2018) demonstrated that decidual lymphatics and veins are the first to be invaded by human extravillous trophoblasts in early pregnancy, before the remodeling of spiral arteries, implicating a role in the pathogenesis of PE (He et al. 2017).

In pregnancy, the increased plasma volume and blood flow to placental lakes requires the development of a rich lymphatic network in the decidua (fetal and maternal) to ensure homeostasis. There is a variation in lymphatic vessel architecture, and evidence for placental bed lymphangiogenesis, during gestation (Volchek et al. 2010). The size and number of blood and lymphatic vessels increases, and blood flow within the uterus rises to 500–600 mL/minute, of which 80% is directed toward the placental bed (Newton and May 2017). In term pregnancies, the lymphatic vessel profiles are large with a prominent open lumen, while in PE, there are reports of an upregulation of lymphangiogenesis (Volchek et al. 2010). These studies, however, did not take into consideration the current classification of the subcategories of early-onset preeclampsia (EOPE) and late-onset preeclampsia (LOPE) (Brown et al. 2018).

Human immunodeficiency virus (HIV) infection contributes significantly to maternal and fetal morbidity and mortality worldwide (Ghulmiyyah and Sibai 2012; Moodley et al. 2016). More than 17 million women are infected with HIV globally, of whom the majority live in the sub-Saharan region (Chau et al. 2017). Currently, 1.2 million women who are pregnant are infected with HIV globally (Global AIDS Monitoring 2023 (UNAIDS 2022); UNAIDS 2023 estimates). The leading cause of maternal mortality rate (MMR) in South Africa (SA) is non-pregnancy-related infections, such as HIV, tuberculosis, pneumonia, etc. (National Department of Health Annual Report for 2021). In 2020/2021, the maternal mortality rate (MMR) in South Africa (SA) was 120.9 maternal deaths per 100,000 live births. This represents a slight decrease in the period 2021/2022, when the MMR in SA was 119.1 deaths per 100,000 live births. Currently, the MMR in SA is 125 deaths per 100,000 live births (National Department of Health Annual Report for 2021; Pillay and Moodley 2024).

Pregnant women with HIV have a higher risk of dying during pregnancy and the postpartum period compared with uninfected pregnant women (Lathrop et al. 2014). Moreover, the risk of preterm birth, low birth weight, small for gestational age fetuses, and stillbirth are greater in women living with HIV (Harris and Yudin 2020). In KwaZulu-Natal Province (SA), the HIV prevalence in pregnant women is 44.5%, and it remains a serious public health concern (Jim and Karumanchi 2017). However, since the introduction of highly active antiretroviral therapy (HAART), mother-to-child transmission of HIV has decreased to < 2% ,and both neonatal and maternal prognosis have improved considerably (Okada et al. 2018). Hypertensive disorders of pregnancy affects 4–8% of all pregnancies worldwide (Booker 2020). Globally, it is also responsible for over 500,000 fetal and neonatal deaths and over 70,000 maternal deaths (Magee et al. 2022). In SA, preeclampsia (PE) and eclampsia account for the majority of deaths associated with hypertensive disorders of pregnancy (Moodley 2020).

The lymphatic system has a vital role in homeostasis, absorption of lipid molecules, and immune cell trafficking (Ho and Srinivasan 2020; Yousef et al. 2021; Arasa et al. 2021). Of note, lymphangiogenesis (growth of new lymphatics) is associated with inflammatory conditions such as viral infections (Deng et al. 2023). There is a reduced placental transfer of maternal antibodies during HIV infection (Dimitriadis et al. 2009). It is not the only pathway used by the virus to infect other cells, such as macrophages and dendritic cells. The dual tropic HIV-1 variants, using the co-receptors CCR5 and CXCR4, can result in infection of monocytes/macrophages, leading to entry into the mucosa and the lymphatic endothelial cells (LECs) (Vinketova et al. 2016). Endothelial cell progenitors for capillary lymphatics originate from the endothelial vein. From the capillary lymphatics, the virus is transported to the lymph nodes, where it continues replicating (Pijnenborg et al. 2010; Ergen et al. 2018). There is evidence of HIV-1 affecting the dysfunction of vascular endothelial blood and lymphatic cells. It has been reported that the HIV-1 envelope glycoprotein (gp120) and its accessory protein transactivator of transcription (Tat) both contribute to vasculopathy. The HIV-1 gp120 induces apoptosis in endothelial cells (Hijmans et al. 2018). Furthermore, the HIV gp120 compromises the lymphatic endothelial barrier by inducing hyperpermeability (Zhang et al. 2012). Slit2 inhibits Robo4 with fibronectin, and then protects gp120, making Slit2/Robo4 a regulator of endothelial permeability (Red-Horse 2008; Zhao 2015).

Recently, data have suggested that HIV proteins may contribute to the production of transforming growth factor beta-1 (TGF-β1) and Tat protein (Wang et al. 2011). The latter has been shown to induce synthesis of TGF-β1 by human leukocytes (Wang et al. 2011). HIV infection, with its S75X variant p17, is reported to promote angiogenesis and lymphangiogenesis (Volchek et al. 2010; He et al. 2017), and it has been observed that the HIV-1 Tat protein induces production of proinflammatory cytokines by human dendritic cells and monocytes/macrophages through engagement of the TLR4–MD2–CD14 complex and activation of the NF-κβ pathway (Newton and May 2017). These proinflammatory cytokines enhance lymphangiogenesis in normal pregnancy and in other organs (Coelho et al. 2006; Newton and May 2017). Furthermore, researchers have observed important lymphangiogenesis activity in the decidua (Tammela and Alitalo 2010; Liao et al. 2012; Brown et al. 2018).

Despite the reduction in vertical transmission of HIV and the decline in maternal mortality rates, highly active antiretroviral therapy (HAART) has been implicated in the regulation of angiogenesis and lymphangiogenesis with endothelium dysfunction (Vranova and Halin 2014; Yu et al. 2014; Veerbeek et al. 2015). Therefore, we hypothesize that HIV infection may interact with the developmental architecture and function of lymphatic capillaries in the peripheral and at the maternal–fetal interface (placental bed) during placentation or during the pregnancy.

The aim of this study was to evaluate lymphatic microvessel density (LMVD) and the field area of these lymphatics within the placental bed in samples obtained from cases of PE, stratified by gestational age into early-onset and late-onset PE, and normotensive controls stratified by HIV status.

## Materials & methods

This study was conducted at a large regional hospital in Durban, South Africa. Ethical approval was obtained from the Biomedical Research Ethic Committee of University of KwaZulu-Natal (reference no. BE 040/12).

Following informed written consent, 87 women undergoing elective caesarean births participated in the study. They were divided into three groups: normotensive (N, *n* = 28), early-onset preeclampsia (EOPE, *n* = 31), and late-onset preeclampsia (LOPE, *n* = 28). Each subgroup was stratified according to HIV status, namely HIV uninfected (HIV −) and HIV infected (HIV +). The healthy normotensive group included women at 38 weeks gestation having a planned caesarean birth. Preeclampsia was defined as new onset of hypertension (> 140/90 mmHg) after 20 weeks of gestation in a previously normotensive patient, associated with proteinuria and or evidence of organ involvement (Magee et al. 2022). Early-onset preeclampsia was defined as hypertension and proteinuria that developed prior to 33 weeks plus 6 days gestational age, while the definition of LOPE was the development of hypertension and proteinuria after 34 weeks.

All HIV-infected women were on HAART, which at the time of obtaining the placental bed samples was a regimen of HAART initiated before pregnancy or during pregnancy. Two regimens were prescribed, i.e., regimen 1: nevirapine/lamivudine/tenofovir and regimen 2: tenofovir/emtricitabine/efavirenz. The inclusion criteria consisted of specimens representative of arteriole and lymphatic capillaries, specimens representative of decidua and myometrium, and a specified HAART regimen.

### Placental bed collection

Placental bed biopsies were collected at the time of caesarean delivery (CD) according to the technique described by Pijnenborg et al. (2010) and Veerbeek et al. (2015). Following the birth of the baby and delivery of the placenta, a central wedge of placental bed, 1 cm^3^ in diameter, was biopsied.

The placental bed biopsy was fixed in 10% buffered formaldehyde for 1 h, dehydrated, and embedded in paraffin wax. Three-micron sections were stained with hematoxylin and eosin (H&E) to confirm the validity of a true placental bed specimen by identification of spiral arteries together with extravillous trophoblast cells.

### Immunohistochemistry

Sections (3 µm) were immunostained using the Dako Envision Flex detection system kit (Envision + System + HRP; K800021; Dako, Denmark) with a monoclonal mouse anti-human podoplanin (PDPN) antibody (ready-to-use; clone D2-40; 20 min; Dako, Germany). The lymph node served as a positive control. Replacement of the primary antibody with a buffer or with nonimmune sera of the same IgG class served as a method control and IgG control, respectively. A qualitative evaluation of PDPN immunolocalization was performed.

### Microvessel morphometry

Serial sections of H&E- and PDPN-stained placental biopsy samples, consisting of decidua and myometrium, were analyzed. A qualitative assessment of arteries and LMVs were reported according to their size, shape, and density.

Only sections identifying decidua, myometrium, lymphatic microvessels, and spiral arterioles/veins were retained for analysis. The analysis quantified microvessels on tissue stained with PDPN at 20× using the Axioscope A1 microscope (Carl Zeiss, Germany) interfaced with the Axiovision image analysis software (version 4.8.3; Carl Zeiss, Germany).

### Lymphatic microvessel density

The inclusion criterion was the presence of a LMV immunostained with PDPN at an initial magnification of 20×. All blood vessels were excluded. The number of vessels per region of interest (ROI) within the decidua and myometrium was assessed. The LMVD was calculated from the number of vessels per ROI in square microns viewed (*n*/μm^2^). Lymphatic microvessels were also categorized by size into small, medium, and large within the decidua and the myometrium. An average ratio of 1 spiral artery to 3–5 LMVs within the decidua was viewed as normal (Red-Horse 2008). A correlation between the LMVD and pregnancy outcomes [severity of the disease, fetal intrauterine growth restriction (IUGR), and baby and placental weight] was evaluated.

### Lymphatic microvessel lumen area

After outlining the external lymphatic endothelium cells (LEC), the internal area of the lymphatic capillary (ALC) lumen was calculated with software and expressed in µm^2^. The mean area of lymphatic capillary lumens (μm^2^), i.e., the average of all the vessel areas in square microns, was determined. The ALC was considered as “small” if the area was < 5000 µm^2^ (including collapsed lymphatic capillaries) and “large” if the area was ≥ 5000 µm^2^. LMVD and LMV luminal area were evaluated within the decidua and myometrium according to pregnancy status (PE and normotensive). We classified the capillaries into “small” and “large” on the basis of capillary diameters, which were 50–200 μm (Scallan et al. 2010).

### Statistical analysis

Data were analyzed using SPSS statistical software (version 25 for Windows; IBM Corp., USA). Post-normality testing, parametric data are presented as means and standard deviations. Subgroup comparisons were done using Mann–Whitney *U* tests to compare two groups, and Dunn’s multiple comparison test was used to compare more than two groups. A value of *p* < 0.05 was considered significant.

## Results

The associations between demographic/clinical data across study groups is presented in Table 1.

**Table 1:** Demographics of the preeclampsia and normotensive groups (*n* = 87)

Parameters	Study group			*p*-Value		
	N (*n* = 28)	EOPE (*n* = 31)	LOPE (*n* = 28)	N versus EOPE	N versus LOPE	EOPE versus LOPE
Maternal age, mean (years)	27.1 ± 5.4	32.56 ± 3.48	37.63 ± 3.48	*p* = 0.001	*p* = 0.1	*p* = 0.01
Gestational age (weeks)	38.5 ± 1.1	32.56 ± 3.48	37.63 ± 3.48	*p* = 0.01	*p* = 0.1	*p* = 0.01
Maternal systolic BP (mmHg)	110.52 ± 8.75	155.2 ± 2.82	160.5 ± 16.49	*p* = 0.001	*p* = 0.001	*p* = 0.05
Maternal diastolic BP (mmHg)	68.14 ± 6.88	99.0 ± 0.98	100.52 ± 17.98	*p* = 0.01	*p* = 0.01	*p* = 0.05
Baby weight (g)	3302.475 ± 257.3	2091.45 ± 98	2961.55 ± 119.73	*p* = 0.001	*p* = 0.05	*p* = 0.001

*N* normotensive, *EOPE* early-onset preeclampsia, *LOPE* late-onset preeclampsia

*p* < 0.05, *p* < 0.01, and *p* < 0.001 show statistical significance

### Immunostaining of PDPN in LMVs

**Fig. 1:**
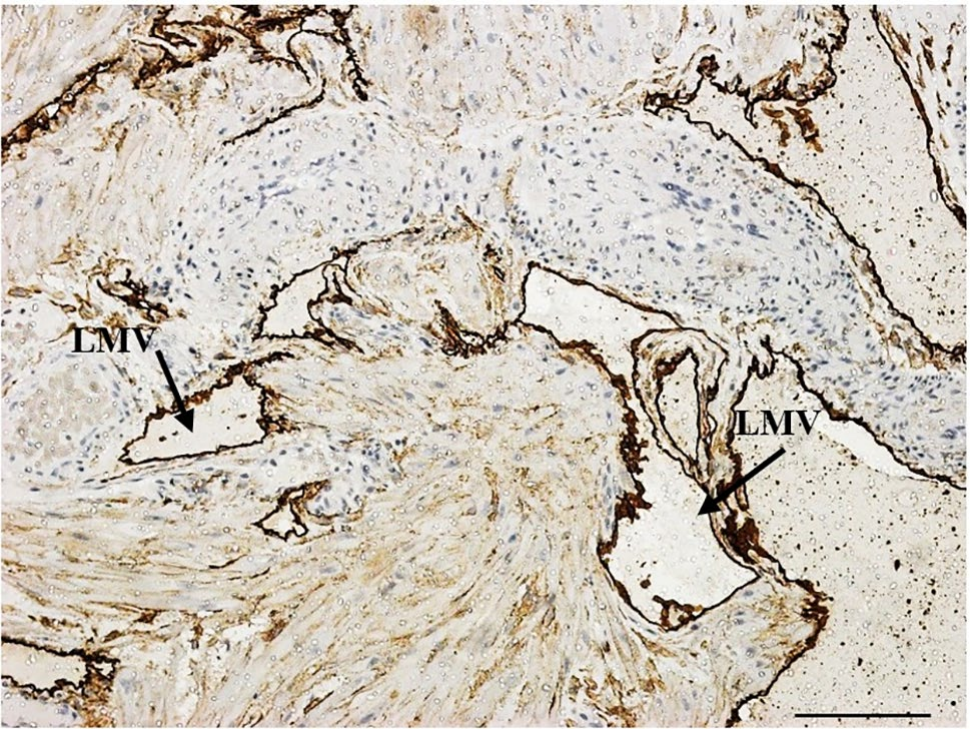
Normotensive HIV-positive: large and small collapsed LMVs were observed mainly in the myometrium as well as at the decidual–myometrial junction. *LMV* lymphatic microvessel. Scale bar = 100 µm

Within the normotensive sample, there was a dense LMV network within the decidua. PDPN was immunolocalized within endothelial cells of LMVs in the decidua and myometrium. Trophoblast cells were not immunostained with PDPN; however, they were identified by structure and size within H&E- and PDPN-immunostained sections. LMVs varied in size and shape. Large LMVs were noted adjacent to spiral arteries. Small and collapsed LMVs were observed mainly in the myometrium, as well as at the decidual–myometrial junction (Fig. 1).

**Fig. 2:**
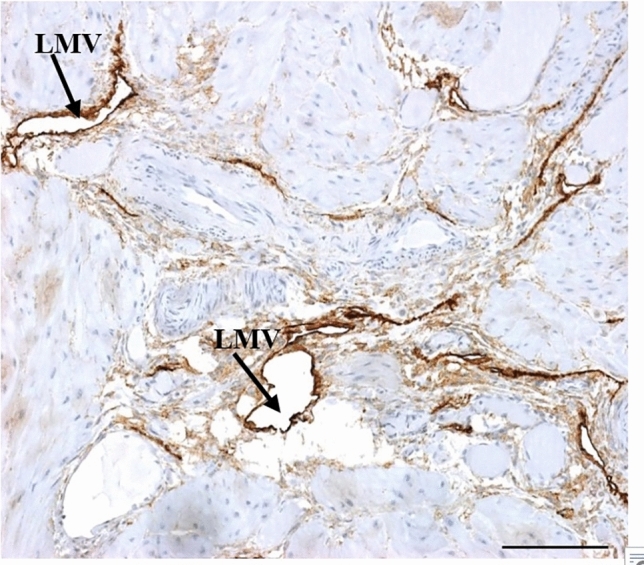
Early-onset preeclampsia HIV-positive: myometrium of early-onset preeclampsia with a collapsed lymphatic microvessel (LMV). Scale bar = 100 µm

However, small and collapsed LMVs occurred predominantly in the EOPE group. Long and collapsed LMVs were frequently observed in the EOPE group. Similarly, the myometrium had a rich network of small and collapsed LMVs (Fig. 2).

**Fig. 3:**
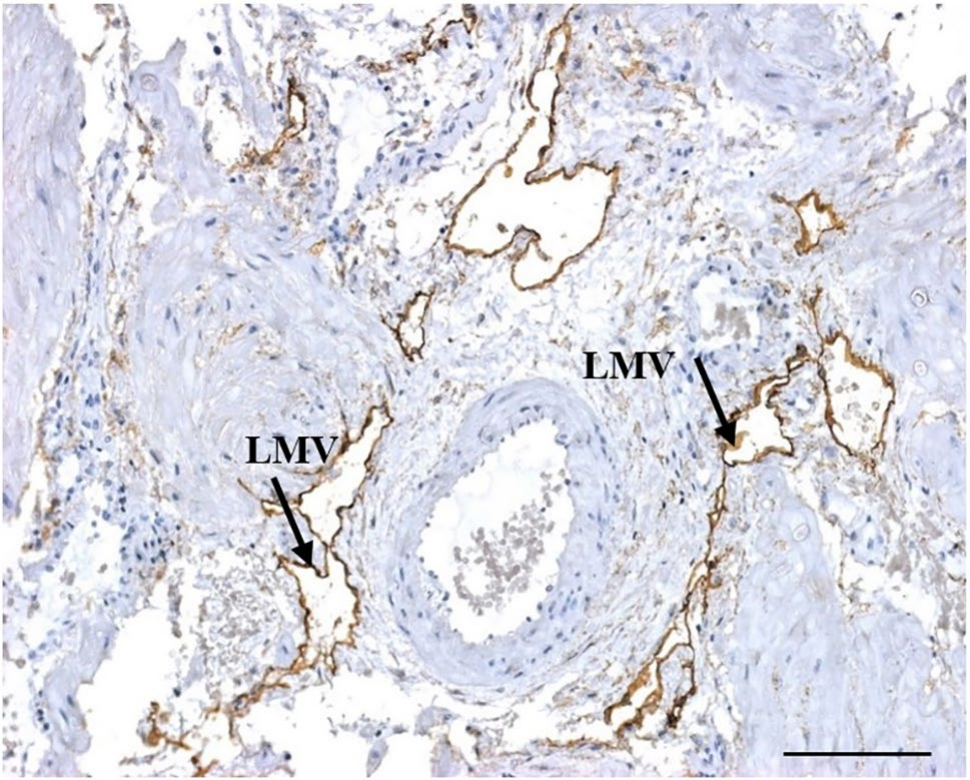
Late-onset preeclampsia HIV-positive: large LMVs within myometrium of late-onset preeclampsia. Scale bar = 100 µm

Large LMVs and large blood vessels occurred in both LOPE and normotensive tissue. In LOPE, abundant infiltrate of polymorphonuclear leukocytes and macrophage-like cells were observed around small and collapsed LMVs (Fig. 3). These are immune cells that include polymorphic multilobed nuclei.

### Morphometric analysis of LMVD and LMV field area within the placental bed in preeclampsia and normotensive

In PE (*n* = 59), the mean LMVD was 7.4 ± 3.0 units compared with a mean of 6.8 ± 3.3 in the normotensive (N) pregnant group (*p* = 0.01). The overall LMV field area per ROI in PE (*n* = 59) and N (*n* = 28) was 13,017.5 ± 247.6 µm^2^ and 5232.1 ± 171.4 µm^2^, respectively (*p* = 0.001).

### Evaluation of LMVD and LMV lumen area within the placental bed in EOPE and LOPE

**Fig. 4:**
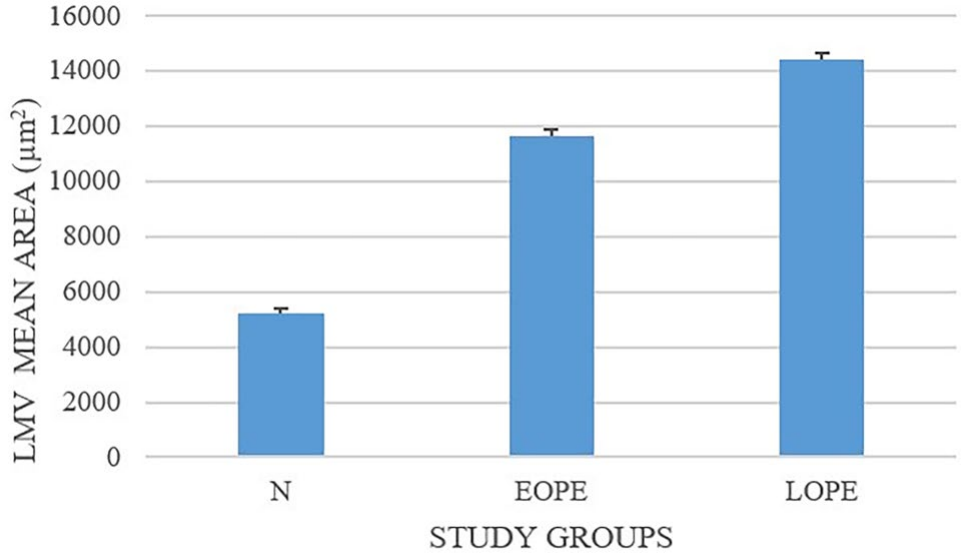
Lymphatic microvessel field area across study groups, irrespective of HIV status

**Fig. 5:**
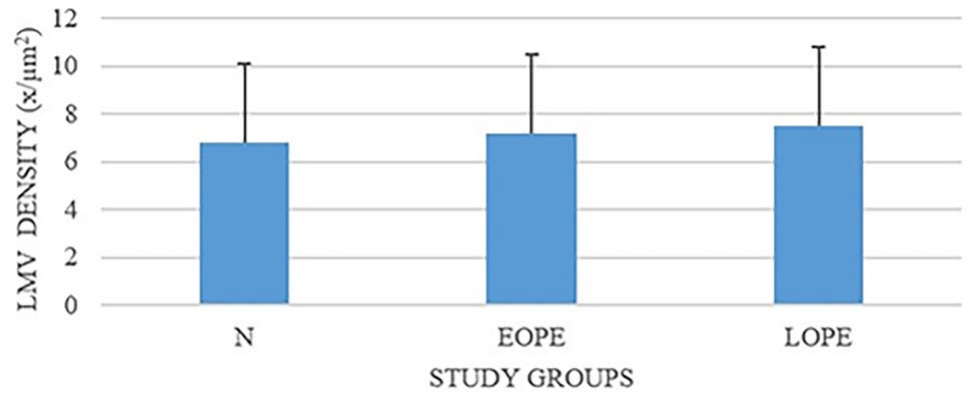
Lymphatic microvessel density across study groups, irrespective of HIV status

The LMV field area (mean ± standard deviation) within the placental bed of EOPE and LOPE was considerably larger compared with the normotensive (N) group (*p* = 0.005 and *p* = 0.0001, respectively). Furthermore, at gestational age of 32.56 ± 3.48 weeks, the EOPE group had a significantly higher LMV lumen area (11,663.8 ± 235.4 µm^2^) compared with the N group (5232.1 ± 171.4 µm^2^) (*p* = 0.0001). At gestational age of 37.63 ± 3.48 weeks, the LOPE group had a higher LMV area (14,416.0 ± 257.7 µm^2^) compared with the N group (5232.1 ± 171.4 µm^2^) (*p* = 0.0001). The distribution of LMVs varied in number and size from small to large in the different study groups. In the EOPE group, the number of LMVs (7.2 ± 3.3) was significantly different compared with the N group (6.8 ± 3.3) (*p* = 0.01). Similarly, the number of LMVs in LOPE (7.5 ± 3.3) was significantly different compared with the N group (6.8 ± 3.3) (*p* = 0.01). No significant difference was noted in the number of LMVs in EOPE (7.2 ± 3.3) versus LOPE (7.5 ± 3.3) groups (*p* = 0.08) (Table 2; Figs. 4 and 5).

**Table 2:** Lymph angiogenesis in placental bed in early-onset preeclampsia and late-onset preeclampsia, and maternal and fetal outcomes

	Study group			*p*-Value		
Parameter	N (*n* = 28)	EOPE (*n* = 31)	LOPE (*n* = 28)	N versus EOPE	N versus LOPE	EOPE versus LOPE
^4^LMV mean field area (µm^2^)	5232.1 ± 171.4	11,663.8 ± 235.4	14,416.0 ± 257.7	*p* = 0.0001	*p* = 0.0001	*p* = 0.005
LMV mean density (*x*/µm^2^)	6.8 ± 3.3	7.2 ± 3.3	7.5 ± 3.3	*p* = 0.01	*p* = 0.01	*p* = 0.08

*N* normotensive, *EOPE* early-onset preeclampsia, *LOPE* late-onset preeclampsia, *LMV* lymphatic microvessel

*p* < 0.05 is considered significant, and *p* < 0.01 and *p* < 0.001 is considered statistically significant

### Correlations between LMVD and level of blood pressure

A weak positive correlation was observed between the LMVD and BP in the EOPE (*r* = 0.2; *p* = 0.06) and the LOPE (*r* = 0.3; *p* = 0.03) groups (Table 3).

**Table 3:** Correlations between LMVD and blood pressure

Correlation	*r*-Value	*p-*Value
LMVD and BP (N)	0.1	0.44
LMVD and BP (EOPE)	0.2	0.06
LMVD and BP (LOPE)	0.3	0.03

Significance denoted by *p* < 0.05

*EOPE* early-onset preeclampsia, *LOPE* late-onset preeclampsia, *N* normotensive, *LMVD* lymphatic microvessel density, *BP* blood pressure

### Comparison of lymphatic capillary density means

**Fig. 6:**
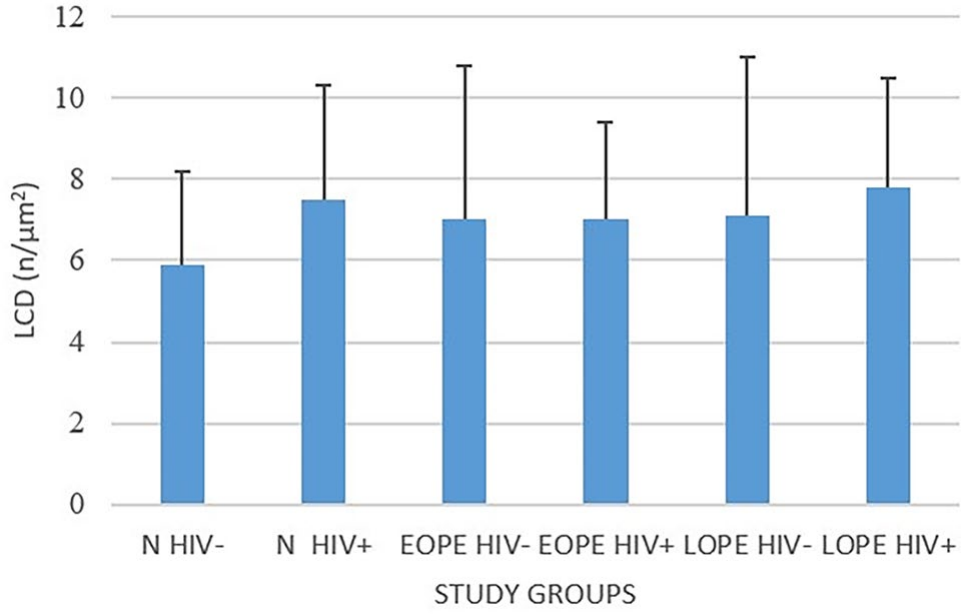
Lymphatic capillary density across the study population

Comparing the lymphatic capillary density (LCD) means in the N− (5.9 ± 2.3) and N+ (7.5 ± 2.8) subgroups, we noticed that HIV infection enhanced LCD lymphangiogenesis in the placental bed of normotensive pregnant women (*p* = 0.01). Furthermore, a similar result was observed between LOPE− and LOPE+ groups (*p* = 0.01). We also report a higher LCD in EOPE− (7 ± 3.8) and in EOPE+ groups (7 ± 2.4) compared with N− (5.9 ± 2.3) (*p* = 0.04). However, there was no significance difference between the two EOPE subgroups (*p* = 0.5) (Tables 4, 5; Fig. 6).

**Table 4:** Lymphatic capillary (LC) density and area lumen capillary (ALC) in pregnancy type HIV− and HIV+

Parameter	Study group					
	N (*n* = 28)		EOPE (*n* = 31)		LOPE (*n* = 28)	
	N HIV−	N HIV+	EOPE HIV−	EOPE HIV+	LOPE HIV−	LOPE HIV+
LCD mean + SD (*n*/µm^2^)	5.9 ± 2.3	7.5 ± 2.8	7 ± 3.8	7 ± 2.4	7.1 ± 3.9	7.8 ± 2.7
ALC, mean ± SD (µm^2^)	4629.3 ± 112.9	6643.8 ± 192.1	6079 ± 240.5	6029.4 ± 412.9	6118.5 ± 193.0	12,527.9 ± 225.2
Total slides viewed	14	14	15	16	14	14

*LCD* lymphatic capillary density, *ACL* area lymphatic capillary lumen, *n* number. Differences were considered significant at *p* < 0.05

**Table 5:** *p*-Values

Groups compared	*p*-Value	
	LCD	ALC
N− versus N+	0.1	0.05
N− versus EOPE−	0.04	ns
N− versus EOPE+	0.04	ns
EOPE+ versus EOPE−	0.5	0.5
LOPE− versus LOPE+	0.01	0.001

### Comparison of mean area of lymphatic capillary lumen

A significant difference was noted at *p* < 0.05.

N− versus N+, *p* = 0.05; EOPE− versus EOPE+, *p* = 0.5; LOPE− versus LOPE+, *p* = 0.001.

**Fig. 7:**
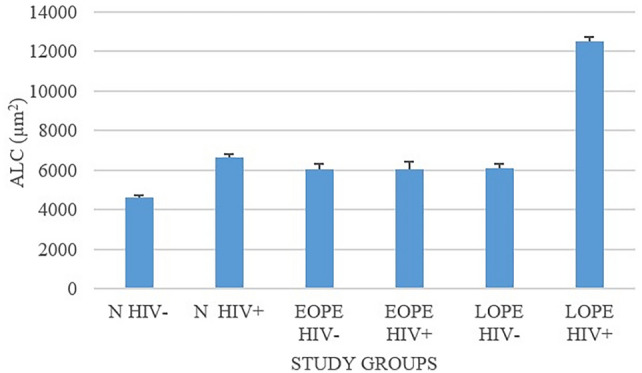
Mean area of the lymphatic capillary lumen across the study population

In the N group, the mean area of LC lumen in N+ (6643.8 ± 192.1) was enhanced and significantly different from N− (4629.3 ± 112.9) (*p* = 0.05). The mean ALC in LOPE− was smaller than in LOPE+ (6118.5 ± 193.0 versus 12,527.9 ± 225.2; *p* = 0.001). We observed no statistical differences between mean ALCs of EOPE− and EOPE+ groups (6079 ± 240.5 versus 6029.4 ± 412.9; *p* = 0.5) (Tables 4and 5; Fig. 7).

**Fig. 8:**
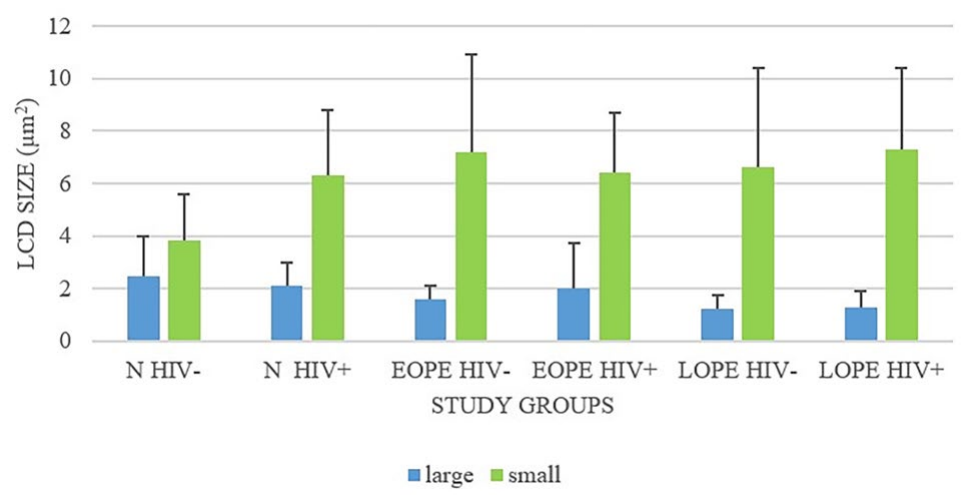
Lymphatic capillary density for large and small lymphatic microvessels

HIV negative status seems to make a statistical difference between large (L) and small (S) capillaries in the N and LOPE groups (*p* = 0.06 and *p* = 0.03, respectively). LC were predominately in the myometrium. No significant difference was noted between mean LCD in the decidua (7 ± 1.6) and LCD in the myometrium (4.6 ± 2.4) in the N− group (*p* = 0.1). The distribution of LC in N+ was more in the decidua (9 ± 2.9) compared with the myometrium (6.0 ± 2.0; *p* = 0.05) (Tables 6and 7; Fig. 8).

**Table 6:** Lymphatic capillary density according to size

Parameters	Study groups							
	^3^N (*n* = 28)		^1^EOPE (*n* = 31)			^2^LOPE (*n* = 28)		
	N HIV −	N HIV +		EOPE HIV −	EOPE HIV +		LOPE HIV −	LOPE HIV +
S ≤ 5000µm^2^	3.8 ± 1.8	6.3 ± 2.5		7.2 ± 3.7	6.4 ± 2.3		6.6 ± 3.8	7.3 ± 3.1
L ≥ 5000µm^2^	2.5 ± 1.5	2.1 ± 0.9		1.6 ± 0.5	2 ± 1.7		1.25 ± 0.5	1.3 ± 0.6

*L* large lymphatic capillary, *S* small lymphatic capillary.

Significance denoted by*p*  < 0.05.

HIV negative status seems to make a statistical difference between L and S in the N and LOPE groups (*p* = 0.06 and *p* = 0.03, respectively)

**Table 7:** Lymphatic capillary density *p* values comparison of L versus S within groups

Comparison of L versus S within groups	
Parameter	*p*-Value (L versus S)
N−	0.06
N+	0.5
EOPE−	0.4
EOPE+	0.6
LOPE−	0.03
LOPE+	0.2

### Antiretroviral therapy

Among the normotensive pregnant women with HIV, 1 patient was on regimen 1 and 13 were on regimen 2. In the subgroup EOPE+, ten participants were on regimen 1 and four participants were on regimen 2, whereas six and eight participants were on regimen 1 and regimen 2 in LOPE+, respectively. The comparison between LOPE+ (8 cases) and N+ (13 cases) on regimen 2 showed a significant increase of lymphangiogenesis in LOPE+ specimens (*p* = 0.05). Tenofovir was the common denominator antiretroviral used in all infected subgroups. Regimens 1 and 2 were predominately used in EOPE+ and in LOPE+, respectively. The two regimens were almost equally prescribed in LOPE.

## Discussion

This study is novel in that it demonstrates lymphatic microvessel density and luminal area within the placental bed of normotensive pregnant women versus women with PE (stratified by gestational age into EOPE and LOPE). We report higher LMVD and lymphatic luminal area within the placental bed of PE compared with normotensive pregnant women. More specifically, we demonstrate that LMVD in the placental bed of EOPE and LOPE was dependent on gestational age, and that maternal and fetal outcomes were associated with the level of lymphatic vessel differentiation.

Our findings corroborate the presence of a rich LMV network at the maternal–fetal interface (Wang et al. 2011; Volchek et al. 2010; Liu et al. 2015). Notably, plasma volume and blood flow to placental lakes in pregnancy requires the development of an extensive lymphatic network at the decidua (fetal and maternal) to ensure homeostasis. We report a significant difference in LMVD between normotensive pregnant and PE groups irrespective of gestational age. In normal pregnancy, it is noted that the size and number of lymphatic vessels and blood vessels increase, resulting in blood flow of 500–600 mL/min, with 80% being directed to the placental bed (Newton and May 2017).

Pregnancy is considered a state of mild-to-moderate maternal systemic inflammation in response to the fetal allograft (Mor et al. 2011; Lely et al. 2013; Graham et al. 2017). In response, the placenta produces a range of immunomodulatory hormones and cytokines that promote lymphangiogenesis (Mor et al. 2011; Lely et al. 2013; Vranova et al. 2014; Liu et al. 2015; Veerbeek et al. 2015; Graham et al. 2017). Moreover, in PE, the exaggerated inflammatory milieu promotes an increase of lymphangiogenic factors (Harmon et al. 2016; Aggarwal et al. 2019). Our observation of elevated LMVD and LMV luminal area in PE compared with normotensive pregnancies may be a compensatory mechanism to serve as a conduit to remove the excess fluid (Volchek et al. 2010). Nonetheless, there is controversy on the pathogenesis of this reaction. Lymphangiogenic markers such as VEGF-C and VEGF-D are elevated in PE (Bates 2011; Shange et al. 2017). Similarly, Shange et al. (2017) reported no difference in serum VEGF-C expression between PE and normotensive pregnancies, Bates (2011) reported very little evidence of the role of VEGF-C in PE, while Andraweera et al. (2012) limited its role to EOPE. Despite the controversy in the level of VEGF-C in PE, the regulation of lymphangiogenesis is complex, and is not only dependent on the level of cytokines (Zampell et al. 2012a, b). Indeed, Rutkoweski et al. (2006) and Goldman et al. (2007), reported a failure in lymphatic regeneration in the presence of VEGF-C (exogenous or overexpression). It seems that, in the heightened proinflammatory state of PE, together with the hypovolemia emanating from the nonphysiological remodeling of spiral arteries, lymphangiogenesis is upregulated to facilitate collection of extravagated fluid. Cytokines responsible for the upregulation of lymphangiogenesis, such as hepatocyte growth factor (HGF), fibroblast growth factor (FGF2), interleukin-1 beta and interleukin-6 (IL-1B; IL-6), and tumor necrosis factor (TNF-α), are also found to be upregulated in PE (Zampell et al. 2012a, b).

In PE, HIF-1α gene expression is upregulated, creating a hypoxic microenvironment (Rath et al. 2016). Furthermore, HIF-1α plays a coordinating role of lymphangiogenesis in the wound repair process (Zampell et al. 2012a, b). Therefore, we suggest that the increased lymphangiogenesis observed in the placental bed of PE may be the result of HIF-1α upregulation.

Moreover, we report an upwards trend of LMVD in the placental bed of LOPE compared with EOPE, albeit nonsignificant. Red-Horse et al. (2008) reported the presence of increasing lymphatic vessels in the decidua at different gestational ages: first trimester 10, second trimester 12, and third trimester 13 for normal pregnancy. Notably, we report a lower mean LMVD at term compared with the latter author (Red-Horse et al. 2008). At this stage, it is plausible that VEGF-C and VEGFR-3 immunolocalization at the placental bed and the need for further upregulation of lymphangiogenesis are not required, which may explain our findings.

Coelho et al. (2006) reported a decrease of microvessel arterioles in severe PE, although they did not consider the recent EOPE and LOPE classification by the International Society for the Study of Hypertension in Pregnancy (ISSHP) (Brown et al. 2018). Their observations, and the findings of our study, indicate that an upregulation of LMVD is reactional to achieve the homeostasis and transport role. Gomanthy et al. (2018) reported a significant prevalence of severe PE among patients with EOPE (Gomanthy et al. 2018).

As expected, we report a significant difference in blood pressure levels between normotensive pregnant and PE groups (*p* = 0.001). The highest maternal BP, particularly in LOPE, was associated with raised lymphangiogenesis in the placental bed. Lopez-Gelston et al. (2018) reviewed the impact of lymphatics in hypertension and considered them to play a role in the kidneys (Lopez-Gelston et al. 2018).

The second main finding of our study was an increase in luminal mean area of LMV within the placental bed of PE (EOPE and LOPE) compared with the control group. The true mechanism regulating lumen formation is still unknown. These LMVs constitute a unidirectional system in which numbers and dimensions respond to the need (Schwartz et al. 2012). Their development and function are modulated by genes interacting mainly at the venous valves (Bazigou et al. 2011). VE-cadherin has been implicated in the pathophysiology of PE; it is overexpressed in the syncytiotrophoblast of LOPE compared with EOPE, while decreased in control groups (Groten et al. 2010). More recently, Yang et al. (2019) reported on the role of VE-cadherin in the formation and maintenance of lymphatics by regulating mechanotransduction and endothelial permeability. Our findings may be the result of exaggerated inflammation in PE with strong lymphangiogenesis density, and limited lumen dimensions, as PE has a reduced volume (due to non-remodeled spiral arteries) with resultant low extravagated fluid.

This novel study also demonstrates the impact of HIV infection and HAART on the LCD and their luminal area within the placental bed. The main observations were the upregulation of lymphangiogenesis associated with the HIV infection in all type of pregnancies. HIV infection, like other inflammatory conditions and viral infections, upregulates lymphangiogenesis (Coelho et al. 2006; Vinketova et al. 2016). Lymphangiogenesis is enhanced under the direct or indirect action of proinflammatory cytokines (Liao et al. 2012). Our results confirm previous reports where virus infections or other infections or inflammatory conditions increase lymphangiogenesis (Lely et al. 2013; Harmon et al. 2016), with the normotensive pregnant women with HIV showing an upregulation of lymphangiogenesis. Proinflammatory cytokines responsible for increases in lymphangiogenesis (TNF-α, IL-1β, IL-2, and IL-4) (Lely et al. 2013; Aggarwal et al. 2019) were also reported to be significantly elevated in the sera of pregnant women with HIV on HAART (Rusterholz et al. 2007). These cytokines at the placental bed site could be responsible for stimulation of lymphangiogenesis activity.

Increased lymphangiogenesis in both EOPE HIV-negative and EOPE HIV-positive groups was observed without significant difference. Nevertheless, we suggest that the HAART might have an impact on vascular endothelial cell growth, repair , and dysfunction. Women with EOPE had been on HAART before pregnancy, and that could improve endothelial dysfunction. A reduction of cytokines was observed 3 months after initiation of treatment with HAART (Veerbeek et al. 2015). They also noticed that only IFN-gamma and IL-mRNA were not influenced by the initiation of HAART. Furthermore, Osuji et al. (2018) found that TNF-α and TGF-β remain significantly elevated even after 12 months of therapy, while IFN-γ remains significantly reduced after 12 months of HAART therapy (Veerbeek et al. 2015). The ARVs may play an antiinflammatory role and inhibit lymphangiogenesis (Shange et al. 2017). The explanation for the lymphangiogenesis in EOPE and LOPE may emanate from the exaggerated inflammation linked to PE (Lely et al. 2013). The proinflammatory cytokines, each to different degrees, contribute to the mechanism of PE pathogenesis (Bates 2011; Andraweera et al. 2012).

The impact of HAART was difficult to assess as we have multiple drug regimens. However, recent data showed that nucleoside/nucleotide reverse transcriptase inhibitors (NRTIs), such as zidovudine or AZT, lamivudine or 3-TC, emtricitabine, and tenofovir or TFD, attenuate angiogenesis and lymphangiogenesis by inducing mitochondrial oxidation stress, impairing the receptors tyrosine kinase (RTK) and restraining the endocytosis of RTK into early endosomes (Parsons-Wingerter et al. 2006). Of note, TFD has little interaction with endothelial dysfunction (Rutkowski et al. 2006; Zampell et al. 2012a). Efavirenz, a non-NRTI largely used in our cohort, has been associated with increasing vessel permeability by alteration of endothelial cell–cell junction characteristics that influence the size of the lumen (large or small) (Goldman et al. 2005); nonetheless, we observed no difference in HIV-infected subgroups. Also, EFV increases cell oxidation and impairs acetylcholine-mediated relaxant response, and increases apoptosis and necrosis of endothelial cells (Rutkowski et al. 2006).

Additionally, HIV-1 proteins may contribute to the production of TGF β-1, which downregulates the proinflammatory response of macrophages, immunosuppresses T cells, and regulates dendritic cell response (Goldman et al. 2005; Wang et al. 2011; Zampell et al. 2012a, b). The HIV-1 Tat protein has been shown to induce synthesis of TGF-β1 by human leukocytes (Wang et al. 2011). Another HIV-1 accessory protein, Nef, causes endothelial activation and dysfunction, elevated apoptosis, ROS generation, and release of monocyte attractant protein-1 (MCP-1) (Goldman et al. 2005; Rath et al. 2016). The effects of ARVs on the endothelial cell is not fully understood and depend on many factors, such as the nature of ARV regimes and the duration of the treatment; however, it is important to note that it does not differentiate between vascular or lymphatic endothelial cells (Gomathy et al. 2018). In addition, in this study, birth weight was associated with increased lymphangiogenesis, possibly reflecting a compensatory measure to maintain homeostasis.

### Limitations and perspectives

Placental bed biopsy is an invasive procedure, hence the small sample size. This study was limited to the immunoexpression of the PDPN marker but could be extended to other markers and genes.

New perspectives in the implication of lymphangiogenesis, trophoblast implantation, and hypertension in PE, as well as the lymphatic immune transport role with perinatal outcomes, are needed.

## Conclusions

This study demonstrates an upregulation of lymphatic microvessel density and lymphatic luminal area in PE compared with normotensive pregnant women. PE is characterized by an exaggerated inflammatory response and a lack of spiral artery transformation, with an expected reduction in extravagated fluid. It is plausible that a compensatory mechanism to the hyperinflammatory reaction may be a dilatation of lymphatic luminal area, or increases in microvessel density, to remove the resultant edema associated with PE development. We observed an increase of lymphatic capillary density and lumen area in the placental bed of women with HIV infection. HAART may play a role in the lymphangiogenesis of EOPE. There were no cumulative responses of HIV infection and PE on lymphangiogenesis within the placental bed.

## Data Availability

No datasets were generated or analyzed during the current study.

## References

[CR1] Aggarwal R, Jain AK, Mittal P, Kohli M, Jawanjal P, Rath G (2019) Association of pro-and anti-inflammatory cytokines in preeclampsia. J Clin Lab Anal. 10.1002/jcla.22834 30666720 10.1002/jcla.22834 PMC6528584

[CR2] Andraweera P, Dekker G, Laurence J, Roberts C (2012) Placental expression of VEGF family mRNA in adverse pregnancy outcomes. Placenta 33(6):467–472 22386962 10.1016/j.placenta.2012.02.013

[CR3] Arasa J, Collado-Diaz V, Halin C (2021) Structure and immune function of afferent lymphatics and their mechanistic contribution to dendritic cell and T cell trafficking. Cells 10(5):1269 34065513 10.3390/cells10051269 PMC8161367

[CR4] Bates DO (2011) An unexpected tail of VEGF and PlGF in pre-eclampsia. Portland Press 10.1042/BST20110671 PMC3399770 22103490

[CR5] Bazigou E, Lyons OT, Smith A, Venn GE, Cope C, Brown NA et al (2011) Genes regulating lymphangiogenesis control venous valve formation and maintenance in mice. J Clin Investig 121(8):2984–2992 21765212 10.1172/JCI58050 PMC3223924

[CR08] Booker WA (2020) Hypertensive disorders of pregnancy. Clin Perinatol 47:817–833 10.1016/j.clp.2020.08.011 33153664

[CR6] Brown MA, Magee LA, Kenny LC, Karumanchi SA, McCarthy FP, Saito S et al (2018) Hypertensive disorders of pregnancy: ISSHP classification, diagnosis, and management recommendations for international practice. Hypertension 72(1):24–43 29899139 10.1161/HYPERTENSIONAHA.117.10803

[CR7] Chau K, Hennessy A, Makris A (2017) Placental growth factor and pre-eclampsia. J Hum Hypertens 31(12):782–786 29115294 10.1038/jhh.2017.61 PMC5680413

[CR8] Cifarelli V, Eichmann A (2019) The intestinal lymphatic system: functions and metabolic implications. Cell Mol Gastroenterol Hepatol 7(3):503–513 30557701 10.1016/j.jcmgh.2018.12.002 PMC6396433

[CR9] Coelho TM, Sass N, Camano L, Moron AF, Mattar R, Stávale JN et al (2006) Microvessel density in the placental bed among preeclampsia patients. Sao Paulo Med J 124(2):96–100 16878193 10.1590/S1516-31802006000200009 PMC11060358

[CR10] Deng H, Zhang J, Wu F, Wei F, Han W, Xu X, Zhang Y (2023) Current status of lymphangiogenesis: molecular mechanism, immune tolerance, and application prospect. Cancers 15(4):1169 36831512 10.3390/cancers15041169 PMC9954532

[CR11] Dimitriadis E, Nie G, Hannan NJ, Paiva P, Salamonsen LA (2009) Local regulation of implantation at the human fetal-maternal interface. Int J Dev Biol 54(2–3):313–322 10.1387/ijdb.082772ed 19757390

[CR12] Ergen B, Güzelmeriç K, Yılmazer G, Ergen C, Sakin Ö, Şimşek EE et al (2018) Immunohistochemical analysis of placental microvessel density and spiral artery doppler results in preeclamptic pregnancies. South Clin Istanbul Eurasia. 10.14744/scie.2018.58076

[CR04] Gathiram P, Moodley J (2020) The role of the renin-angiotensin-aldosterone system in preeclampsia: a review. Curr Hypertens Rep 22:1–9 10.1007/s11906-020-01098-2 32893333

[CR13] Ghulmiyyah L, Sibai B (eds) (2012) Maternal mortality from preeclampsia/eclampsia. Seminars in Perinatology, Elsevier 10.1053/j.semperi.2011.09.011 22280867

[CR14] Goldman J, Le TX, Skobe M, Swartz MA (2005) Overexpression of VEGF-C causes transient lymphatic hyperplasia but not increased lymphangiogenesis in regenerating skin. Circ Res 96(11):1193–1199 15890974 10.1161/01.RES.0000168918.27576.78

[CR15] Gomathy E, Akurati L, Radhika K (2018) Early-onset and late-onset preeclampsia-maternal and perinatal outcomes in a rural tertiary health center. Int J Reproduct Contracept Obstet Gynecol 7(6):2266–2269

[CR16] Graham C, Chooniedass R, Stefura WP, Becker AB, Sears MR, Turvey SE et al (2017) In vivo immune signatures of healthy human pregnancy: inherently inflammatory or anti-inflammatory? PLoS ONE 12(6):e0177813 28636613 10.1371/journal.pone.0177813 PMC5479559

[CR17] Groten T, Gebhard N, Kreienberg R, Schleussner E, Reister F, Huppertz B (2010) Differential expression of VE-cadherin and VEGFR2 in placental syncytiotrophoblast during preeclampsia–New perspectives to explain the pathophysiology. Placenta 31(4):339–343 20167365 10.1016/j.placenta.2010.01.014

[CR05] Guo B, Dong R, Liang Y, Li M (2021) Haemostatic materials for wound healing applications. Nat Rev Chem 5(11):773–791 10.1038/s41570-021-00323-z 37117664

[CR18] Harmon AC, Cornelius DC, Amaral LM, Faulkner JL, Cunningham MW, Wallace K et al (2016) The role of inflammation in the pathology of preeclampsia. Clin Sci 130(6):409–419 10.1042/CS20150702 PMC5484393 26846579

[CR19] Harris K, Yudin MH (2020) HIV Infection in Women: A 2020 Update. Prenat Diagn. 10.1002/pd.5769 33405240 10.1002/pd.5769

[CR20] He N, van Iperen L, de Jong D, Szuhai K, Helmerhorst FM, van der Westerlaken LA et al (2017) Human extravillous trophoblasts penetrate decidual veins and lymphatics before remodeling spiral arteries during early pregnancy. PLoS ONE 12(1):e0169849 28081266 10.1371/journal.pone.0169849 PMC5230788

[CR21] Hijmans JG, Stockleman K, Reiakvam W, Levy MAV, Brewster LM, Bammert TD, Greiner JJ, Connick E, DeSouza CA (2018) Effects of HIV-1 gp120 and tat on endothelial cell sensescence and senescence-associated micro RNAs. Physiol Rep 6(6):p137 10.14814/phy2.13647 PMC5875545 29595877

[CR22] Ho YC, Srinivasan RS (2020) Lymphatic Vasculature in Energy Homeostasis and Obesity. Front Physiol 11:3 32038308 10.3389/fphys.2020.00003 PMC6987243

[CR23] Jim B, Karumanchi SA (2017) Preeclampsia: pathogenesis, prevention, and long-term complications. Semin Nephrol 37(4):386–397 10.1016/j.semnephrol.2017.05.011 28711078

[CR24] Lathrop E, Jamieson DJ, Danel I (2014) HIV and maternal mortality. Int J Gynecol Obstet 127(2):213–215 10.1016/j.ijgo.2014.05.024 PMC4302331 25097142

[CR25] Lely AT, Salahuddin S, Holwerda KM, Karumanchi SA, Rana S (2013) Circulating lymphangiogenic factors in preeclampsia. Hypertens Preg 32(1):42 10.3109/10641955.2012.697953 PMC3570685 22957504

[CR26] Liao W-X, Laurent LC, Agent S, Hodges J, Chen D-b (2012) Human placental expression of SLIT/ROBO signaling cues: effects of preeclampsia and hypoxia. Biol Reprod 86(4):111 22262697 10.1095/biolreprod.110.088138 PMC3338657

[CR27] Liu H, Li Y, Zhang J, Rao M, Liang H, Liu G (2015) The defect of both angiogenesis and lymphangiogenesis is involved in preeclampsia. Placenta 36(3):279–286 25586742 10.1016/j.placenta.2014.12.013

[CR28] Lopez Gelston CA, Balasubbramanian D, Abouelkheir GR, Lopez AH, Hudson KR, Johnson ER et al (2018) Enhancing renal lymphatic expansion prevents hypertension in mice. Circ Res 122(8):1094–1101 29475981 10.1161/CIRCRESAHA.118.312765

[CR29] Magee LA, Brown MA, Hall DR, Gupte S, Hennessy A, Karumanchi SA, Kenny LC, Mccarthy F, Myers J, Poon LC (2022) The 2021 international society for the study of hypertension in pregnancy classification, diagnosis & management recommendations for international practice. Preg Hypertens 27:148–169 10.1016/j.preghy.2021.09.008 35066406

[CR09] Moodley J (2020) Deaths from hypertensive disorders of pregnancy during 2017-2019: declining trends in South Africa. Obstetrics and Gynaecology Forum, In House Publications, 3-5

[CR30] Moodley J, Onyangunga O, Maharaj N (2016) Hypertensive disorders in primigravid black South African women: a one-year descriptive analysis. Hypertens Pregnancy 35(4):529–535 27391770 10.1080/10641955.2016.1193190

[CR31] Mor G, Cardenas I, Abrahams V, Guller S (2011) Inflammation and pregnancy: the role of the immune system at the implantation site. Ann N Y Acad Sci 1221(1):80 21401634 10.1111/j.1749-6632.2010.05938.x PMC3078586

[CR32] Newton ER, May L (2017) Adaptation of maternal-fetal physiology to exercise in pregnancy: the basis of guidelines for physical activity in pregnancy. Clin Med Insights: Women’s Health. 10.1177/1179562X17693224 10.1177/1179562X17693224 PMC5428160 28579865

[CR33] Okada H, Tsuzuki T, Murata H (2018) Decidualization of the human endometrium. Reproduct Med Biol 17(3):220–227 10.1002/rmb2.12088 PMC6046526 30013421

[CR011] Osuji FN, Onyenekwe CC, Ahaneku JE, Ukibe NR (2018) The effects of highly active antiretroviral therapy on the serum levels of pro-inflammatory and anti-inflammatory cytokines in HIV infected subjects. J Biomed Sci 25:1–8 10.1186/s12929-018-0490-9 PMC6276218 30501642

[CR06] Otalike F, Otubu J, Bakut F (2022) Comparison of outcomes in early and late onset preeclampsia in two district hospitals in Abuja. Trop J Obstet Gynaecol 39(1):12–17

[CR34] Parsons-Wingerter P, Mckay TL, Leontiev D, Vickerman MB, Condrich TK, Dicorleto PE (2006) Lymphangiogenesis by blind-ended vessel sprouting is concurrent with hemangiogenesis by vascular splitting. Anatom Record Part a: Discov Mole, Cell, Evolut Biol: off Pub Am Associat Anatom 288(3):233–247 10.1002/ar.a.20309 16489601

[CR35] Pijnenborg R, Brosens I, Romero R (2010) Placental bed disorders: basic science and its translation to obstetrics. Cambridge University Press

[CR07] Pillay Y, Moodley JM (2024) Will South Africa meet the sustainable development goals target for maternal mortality by 2030? S Afr Med J 114(5). 10.7196/SAMJ.2024.v114i5.1802 10.7196/SAMJ.2024.v114i5.1802 39041479

[CR37] Rath G, Aggarwal R, Jawanjal P, Tripathi R, Batra A (2016) HIF-1 alpha and placental growth factor in pregnancies complicated with preeclampsia: a qualitative and quantitative analysis. J Clin Lab Anal 30(1):75–83 25545166 10.1002/jcla.21819 PMC6807228

[CR38] Red-Horse K (2008) Lymphatic vessel dynamics in the uterine wall. Placenta 29(1):55–59 10.1016/j.placenta.2007.11.011 PMC2435487 18155143

[CR39] Rusterholz C, Hahn S, Holzgreve W (eds) (2007) Role of placentally produced inflammatory and regulatory cytokines in pregnancy and the etiology of preeclampsia. Seminars in Immunopathology, Springer 10.1007/s00281-007-0071-6 17621700

[CR40] Rutkowski JM, Boardman KC, Swartz MA (2006) Characterization of lymphangiogenesis in a model of adult skin regeneration. Am J Physiol-Heart Circulat Physiol 291(3):H1402 10.1152/ajpheart.00038.2006 PMC2751590 16648194

[CR03] Scallan J, Huxley VH, Korthuis RJ (2010) Capillary fluid exchange: regulation, functions, and pathology. Morgan & Claypool Life Sciences, San Rafael. PMID: 21452435. 10.4199/C00006ED1V01Y201002ISP003 21452435

[CR41] Schwartz SR, Rees H, Mehta S, Venter WD, Taha TE, Black V (2012) High incidence of unplanned pregnancy after antiretroviral therapy initiation: findings from a prospective cohort study in South Africa. PLoS ONE 7(4):e36039 22558319 10.1371/journal.pone.0036039 PMC3338622

[CR42] Shange GP, Moodley J, Naicker T (2017) Effect of vascular endothelial growth factors A, C, and D in HIV-associated pre-eclampsia. Hypertens Pregn 36(2):196–203 10.1080/10641955.2017.1297821 28524736

[CR43] Tammela T, Alitalo K (2010) Lymphangiogenesis: molecular mechanisms and future promise. Cell 140(4):460–476 20178740 10.1016/j.cell.2010.01.045

[CR01] UNAIDS (2022) Guidance. Global AIDS Monitoring 2023. Indicators and questions for monitoring progress on the 2021 Political Declaration on HIV and AIDS. https://indicatorregistry.unaids.org/sites/default/files/2023-global-aids-monitoring_en.pdf

[CR45] UNAIDS. 2023. Global Hiv & Aids Statistics—Fact Sheet [Online]. Available: https://www.unaids.org/en/resources/fact-sheet [Accessed 17 July 2023].

[CR010] Veerbeek JH, Uiterweer EP, Nikkels PG, Koenen SV, van der Zalm M, Koster MPH, Burton GJ, van Rijn BB, Franx A (2015) Biopsy techniques to study the human placental bed. Placenta 36(8):775–782 10.1016/j.placenta.2015.05.008 26076963

[CR47] Vinketova K, Mourdjeva M, Oreshkova T (2016) Human decidual stromal cells as a component of the implantation niche and a modulator of maternal immunity. J Pregn. 10.1155/2016/8689436 10.1155/2016/8689436 PMC4864559 27239344

[CR48] Volchek M, Girling JE, Lash GE, Cann L, Kumar B, Robson SC et al (2010) Lymphatics in the human endometrium disappear during decidualization. Hum Reprod 25(10):2455–2464 20729537 10.1093/humrep/deq224

[CR49] Vranova M, Halin C (2014) Lymphatic vessels in inflammation. J Clin Cell Immunol. 10.4172/2155-9899.1000250

[CR51] Wang Y, Sun J, Gu Y, Zhao S, Groome LJ, Alexander JS (2011) D2–40/podoplanin expression in the human placenta. Placenta 32(1):27–32 21095001 10.1016/j.placenta.2010.10.014 PMC3062260

[CR02] Windsperger K, Dekan S, Pils S, Golletz C, Kunihs V, Fiala C, Kristiansen G, Knöfler M, Pollheimer J (2017) Extravillous trophoblast invasion of venous as well as lymphatic vessels is altered in idiopathic, recurrent, spontaneous abortions. Hum Reprod 32(6):1208–1217. 10.1093/humrep/dex058 10.1093/humrep/dex058 28369440

[CR52] Yang Y, Cha B, Motawe ZY, Srinivasan RS, Scallan JP (2019) VE-cadherin is required for lymphatic valve formation and maintenance. Cell Rep 28(9):2397–2412 31461654 10.1016/j.celrep.2019.07.072 PMC6743082

[CR53] Yousef M, Silva D, Chacra NB, Davies N, Löbenberg R (2021) The lymphatic system: a sometimes-forgotten compartment in pharmaceutical sciences. J Pharm Pharm Sci 24:533–547 34694988 10.18433/jpps32222

[CR54] Yu J, Zhang X, Kuzontkoski PM, Jiang S, Zhu W, Li DY et al (2014) Slit2N and Robo4 regulate lymphangiogenesis through the VEGF-C/VEGFR-3 pathway. Cell Commun Signal 12(1):25 24708522 10.1186/1478-811X-12-25 PMC4122147

[CR55] Yu X, Wu H, Yang Y, Wang F, Wang YL, Shao X (2022) Placental development and pregnancy-associated diseases. Mater-Fetal Med 4(1):36–51 10.1097/FM9.0000000000000134 PMC12094368 40406576

[CR56] Zampell JC, Aschen S, Weitman ES, Yan A, Elhadad S, Andrade MDB et al (2012a) Regulation of adipogenesis by lymphatic fluid stasis part I: adipogenesis, fibrosis, and inflammation. Plast Reconstr Surg 129(4):825 22456354 10.1097/PRS.0b013e3182450b2d PMC3433726

[CR57] Zampell JC, Yan A, Avraham T, Daluvoy S, Weitman ES, Mehrara BJ (2012b) HIF-1α: coordinates lymphangiogenesis during wound healing and in response to inflammation. FASEB J 26(3):1027–1039 22067482 10.1096/fj.11-195321 PMC3470728

[CR58] Zhang X, Yu J, Kuzontkoski PM, Zhu W, Li DY, Groopman JE (2012) Slit2/Robo4 signaling modulates HIV-1 gp120-induced lymphatic hyperpermeability. PLoS Pathog 8(1):e1002461 22241990 10.1371/journal.ppat.1002461 PMC3252370

[CR59] Zhao, H., 2015. Roles of Slit-Robo Signaling in Pathogenesis of Multiple Human Diseases: HIV-1 Infection, Vascular Endothelial Inflammation and Breast Cancer (Doctoral dissertation, The Ohio State University).

[CR60] Zhou S, Zhao G, Chen R, Li Y, Huang J, Kuang L, Zhang D, Li Z, Xu H, Xiang W, Xie Y (2024) Lymphatic vessels: Roles and potential therapeutic intervention in rheumatoid arthritis and osteoarthritis. Theranostics 14(1):265 38164153 10.7150/thno.90940 PMC10750203

